# Mating with seminal vesicle-excised male can affect the uterus phospholipid fatty-acids composition during implantation in an experimental mouse model

**DOI:** 10.1590/S1677-5538.IBJU.2018.0485

**Published:** 2019-09-02

**Authors:** Amir Fattahi, Zeinab Latifi, Masoud Darabi, Ali Salmassi, Laya Farzadi, Maghsood Shaaker, Amir Mehdizadeh, Tohid Ghasemnejad, Leila Roshangar, Mohammad Nouri

**Affiliations:** 1 Women’s Reproductive Health Research Center, Tabriz University of Medical Sciences, Tabriz, Iran;; 2 Department of Reproductive Biology, Faculty of Advanced Medical Sciences, Tabriz University of Medical Sciences, Tabriz, Iran;; 3 Department of Biochemistry and Clinical Laboratories, Faculty of Medicine, Tabriz University of Medical Sciences, Tabriz, Iran;; 4 Liver and Gastrointestinal Diseases Research Centers, Tabriz University of Medical Sciences, Tabriz, Iran;; 5 Stem Cell Research Center, Tabriz University of Medical Sciences, Tabriz, Iran

**Keywords:** Uterus, Seminal Vesicles, Male

## Abstract

**Purpose:**

No comprehensive information is available about uterus fatty acid (FA) change during implantation period and possible effects of the seminal vesicle secretion on it.

**Materials and Methods:**

In this study, we evaluated FA composition of uterus phospholipids during the implantation period in intact and seminal vesicle-excised (SVX) mated female mice. Forty NMRI female mice were divided into control (mated with intact male) and seminal vesicle excised (SVX)-mated (mated with SVX-male) groups. The phospholipid fatty acids composition was monitored during the first five days of pregnancy using gas chromatography and also implantation rate was evaluated on fifth day of pregnancy.

**Results:**

We found that levels of linoleic acid (LNA) and arachidonic acid (ARA) showed a decreasing trend from the first to the third day of pregnancy and then started to increase on the fourth day and peaked on the fifth day. In contrast, the level of saturated FA (SFA) increased on the second and third day of pregnancy compared to the first (p<0.05) and then decreased on the fourth and fifth. We also found that the seminal vesicle secretion could affect the levels of LNA, ARA, SFA, and PUFA in uterine phospholipids especially on second and third day. Moreover, there was a positive correlation between ARA level and implantation rate in control but not SVX-mated groups.

**Conclusions:**

It can be concluded that several uterus FA that have important roles in early pregnancy could be affected by seminal vesicle secretion.

## INTRODUCTION

In addition to maintaining cell integrity, modulating cell-cell and cell-matrix interactions and signal transduction, cellular membrane is also a crucial source of various lipid mediators. The phospholipid fatty acids (FA) affect the membrane lipid matrix and consequently the mobility, conformation, and function of the membrane proteins (1). On the other hand, successful implantation of the embryo takes place during a specific period known as the window of implantation in which fusion of the blastocyst and uterus cells membranes is an important event. In support of this, essential roles of lipid molecules in mice embryo invasion have been emphasized previously (2). Beneficial effects of various FA (especially polyunsaturated FA, PUFA) on embryo implantation, and maintenance of pregnancy have been also observed (3). Experimental studies have confirmed the importance of FA as energy source for endometrium decidualization which is a necessary step for implantation (4). It has also been suggested that biophysical properties of the uterus and blastocyst membranes, such as bulk lipid fluidity or phospholipid bilayer polarity change in favor of membrane fusion and consequently the embryo implantation (5).

Phospholipids as a main component of the biological membrane are involved in the production of prostaglandins (PG) and related compounds which have a role in inflammatory processes and immune-mediated responses (6). Roles of such compounds in female fertility have been widely described by previous studies [reviewed by Sugimoto et al. (7)]. Also, change in uterine FA has been considered important in maintaining early pregnancy (8). Since membrane FA of uterus cells are the main precursors of uterus PG (8) and there is a growing body of evidence about crucial roles of PG in embryo implantation, any change in FA composition of the uterus phospholipids during the implantation period may affect the implantation and consequently the pregnancy outcome.

Seminal fluid (SF) contains different molecules that interact with epithelial cells in the female reproductive tract and influence expression of various genes and immune system responses and thus prepare the endometrium for implantation (9). It has been demonstrated that in the female mice deprived of contact with the male SF, the fetal loss rate was higher (10). Moreover, it has been seen that SF can induce expression of PG related genes in swine and horse endometrial cell (11). It was reported that one of the mechanisms through which SF is involved in implantation and pregnancy is regulating PG amount in the female genital tract (12). So, uterus phospholipids FA as the main precursors of the PG production can be possibly affected following mating and insemination. However, there is no information available regarding insemination and SF effects on uterus FA.

There is no detailed information about uterus FA changes during the implantation period in mice which takes place in first five days after mating. Moreover, possible effects of the seminal vesicle secretion as the main part of SF on uterus remain to be clarified. To address these issues, we evaluated FA composition of uterus phospholipids during the implantation period in intact and seminal vesicle-excised (SVX) mated female mice.

## MATERIALS AND METHODS

### Animals

Forty female and 16 male adult albino Naval Medical Research Institute (NMRI) two-month-old mice were obtained from RAZI institute, Iran. The average weight of the animals was 20.5±3.4 g. Animals were housed under standard conditions of 25±2ºC temperature, 60–70% humidity with 12 hrs light/dark cycle and received food (standard pellet manufactured by RAZI institute, Iran), and water *ad libitum*. Fatty acid composition of the standard chow pellet is shown in Table-1. All animal procedures were approved by the Animal Ethical Committee of Tabriz University of Medical Sciences (code TBZMED.REC.1394.357).

**Table 1:** Fatty acid composition of standard chow pellet.

Composition	Standard chow
14:0 (%)	1.1
16:0 (%)	40.6
16:1 (%)	1.4
18:0 (%)	6.8
18:1 (%)	28.8
18:2 (%)	19.6
18:3 (%)	1.4
Carbohydrate	58
Protein	28
Fat	14

Data are expressed as percentage (%) of total

After one week of adaptation, the male mice were randomly divided into normal (n=8) and excised seminal vesicle (n=8) groups. To excise the seminal vesicle glands, the male mice were first anaesthetized with an IP injection of ketamine/xylazine solution, 0.1 mL per 10 g body weight [1 mL ketamine (100 mg mL-1) + 0.5 mL of xylazine (20 mg mL-1) + 8.5 mL of saline] and total bilateral removal of seminal vesicles was performed through the posterior abdominal wall. At three weeks after the operation, the female mice were randomly divided into two groups: 1) the control group (n=20) which was allowed to mate with intact male mice and 2) SVX-mated group (n=20) which was allowed to mate with male mice without seminal vesicle glands. For natural mating, three female mice were placed overnight in a separate cage with a male mouse with or without seminal vesicle glands depending on the type of group.

**Figure 1:**
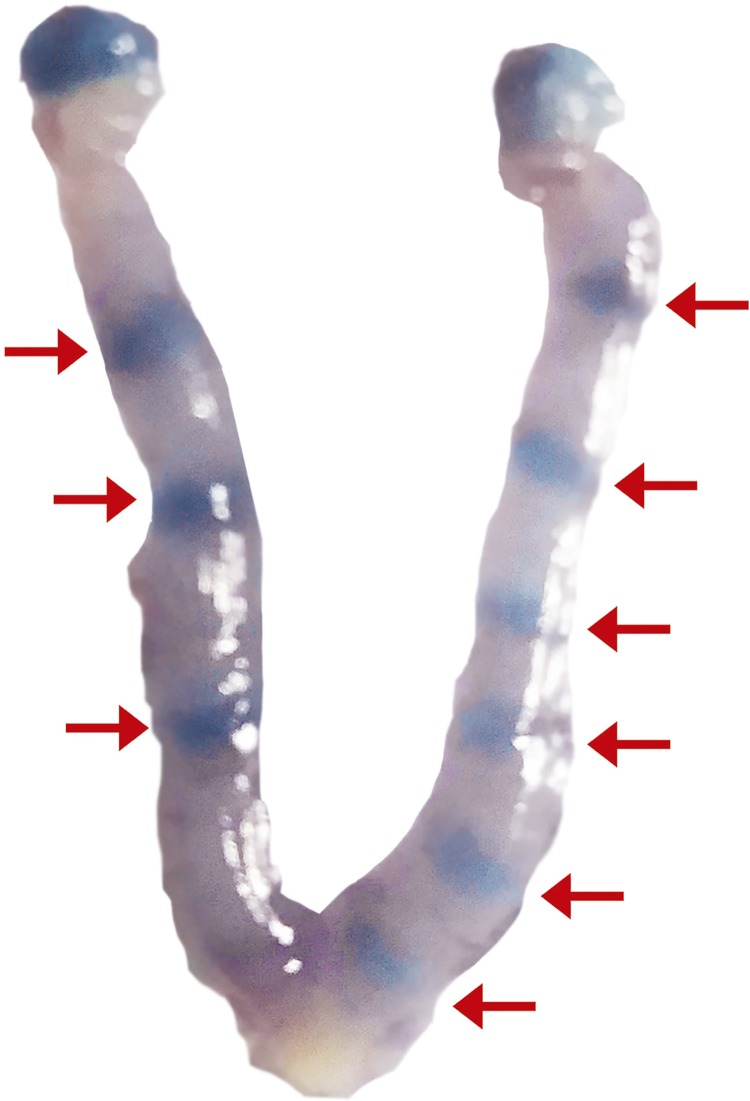
Embryo implantation sites on the mice uterine horns at the fifth day of pregnancy using Chicago blue dye; the arrows indicate implantation.

Observation of a vaginal plug (for control group) or spermatozoa in the vaginal smear (for the SVX-mated group) in next morning indicates day one of pregnancy. The pregnant mice of each group were sacrificed on days 1-5 of pregnancy (four mice for each day of pregnancy). The uterus was surgically removed from each sacrificed female and washed with phosphate buffered saline (PBS). To determine counts of the implantation sites, 0.1 mL of 1% Chicago blue (Sigma Chemical Co., St. Louis, MO) in saline was injected *via* a tail vein based on the previously described method (13). The blue bands on uterine horns were considered as implantation sites (Figure-1) and the number of implantation sites per uterus was considered as implantation rate.

### Fatty acids analysis

The Bligh-Dyer method was used to extract total lipids from the uterine tissues (14). Briefly, tissue was crushed in MeOH/chloroform solution (2:1) and centrifuged. After centrifugation, the supernatant was collected in another tube, chloroform and distilled water were added and the contents were mixed vigorously. The tube was again centrifuged and chloroform part which now contained lipids was collected. The chloroform-lipid fraction was partially dried under nitrogen stream. Thin layer chromatography (TLC) was performed on silica gel plate to separate the phospholipids. In hexane/diethyl ether/glacial acetic acid (70:30:1) solvent system, phospholipids remain in spotting place. After separation, the phospholipid fraction was scraped in a glass tube containing the hexane/methanol solution and methylated tridecanoic acid (13:0, Sigma chemicals) as an internal standard. For esterification of the FA, methanol with acetyl chloride was used as described previously (Lepage and Roy 1986). Fatty acid methyl esters (FAME) were prepared using a one-step direct transesterification. The reaction included a nucleophilic attack of methoxy ion (-CH3O-) to the esteric bonds of between fatty acids and alcohol (glycerol or sphingosine) at 100ºC which led to methyl ester formation. After trans-esterification reaction, 5 mL K2CO3 was added to stop the reaction and separate organic and aqueous phases. FAME located in organic (hexane) phase were collected and the derivatized samples were then injected into a 60×0.25-mm×0.2μM Teknokroma TR CN100 capillary column (Spain) using a Buck Scientific model 610 gas chromatograph (SRI Instruments, Torrance, USA). The oven temperature was increased from 170–210ºC at the rate of 1ºC/min and then maintained stable for 45 minutes. Helium was applied as the carrier gas and the detection was done by flame ionization detector. To determine the retention times of the known FA, standards from Sigma chemicals were injected. Following fatty acids as the main and detectable fatty acids of uterus phospholipid were evaluated: 16:0, 16:1, 18:0, 18:1, 18:2, 18:3, and 20:4. The levels of individual FA were expressed as percentage share of the total. It should be noted that the data for the control group were previously used in a parallel study to evaluate effect of omega-3 and -6 enriched diets (15).

### Statistical analysis

Normal distribution of the data was confirmed by Skewness and Kurtosis tests. To compare the FA levels among various pregnancy days and groups the two-way ANOVA was applied. In the case with significant difference, Tukey’s test was performed as the follow up test. Also, homoscedasticity of the variances was confirmed by Levene’s test. The Pearson correlation test was employed to evaluate possible associations between FA levels and implantation rate (p-values <0.05 were considered significant). SPSS V.16 software was used for the statistical analysis.

## RESULTS

The uterine weight and uterine/body weight ratio of the animals are shown in Table-2. In control group, the uterine weight and uterine/body weight ratio on day one of pregnancy were significantly higher than days two, three and four (p<0.05), but surprisingly these factors did not significantly differ between days one and five of pregnancy. Although wet uterine weight on day five was higher than day two (p<0.05). We observed almost a similar pattern of change in the uterine weight of the SVX-mated group. Although in this group, the uterine/body weight ratio in days four and five of pregnancy were higher than days two and three (*p*<0.05). Besides, the ratios in days four and five were significantly higher in the SVX-mated group compared to the control group (Table-2).

**Table 2:** Animals and their wet uterine weight as well as uterine weight/body weight ratio on various pregnancy days in control (n=20) and seminal vesicle excised (SVX)-mated (n=20) groups.

	Day 1	Day 2	Day 3	Day 4	Day 5
**Control group**					
Body weight (gr)	33.82±3.67	29.80±3.37	33.90±3.04	32.40±2.56	32.70±1.67
Wet uterine weight (mg)	142.25±26.66	70.25±22.06^a^	89.00±12.73^a^	88.33±22.68^a^	115.00±30.47^b^
(Uterine weight/body weight ratio)×1000	4.27±1.14	2.41±0.90^a^	2.54±0.19^a^	2.72±0.43^a^	3.49±0.75^c^
**SVX-mated group**					
Body weight (gr)	31.97±1.62	30.97±0.38	30.22±0.94	32.50±3.54	29.90±3.94
Wet uterine weight (mg)	124.25±26.41	78.75±22.29^a^	75.75±26.86^a^	118.00±6.68^b,c^	139.50±11.12^b, c^
(Uterine weight/body weight ratio)×1000	3.90±0.88	2.55±0.76^a^	2.50±0.85^a^	3.65±0.50^b,c, $^	4.69±0.35^b,c,d, $^

Data are shown as Mean ± S.D; Levene’s test confirmed homoscedasticity of variances and two-way ANOVA Was used to evaluate interaction between groups and days.

Significant difference (p<0.05) in comparison with ^**a**^ Day 1; ^**b**^ Day2; ^**c**^ Day 3 and ^**d**^ Day 4

^**$**^ Significant difference in comparison with the control group

We applied two-way ANOVA to find out if there are significance differences across the two groups or preimplantation days. Our results showed that the levels of palmitoleic acid (16:1), stearic acid (18:0, STA), oleic acid (18:1, OLA) and linolenic acid (18:3) did not significantly change throughout this period (p>0.05, Table-3). However, levels of palmitic acid (16:0, PAM) were significantly lower on day five compared with days two and three of pregnancy (*p*<0.05).

**Table 3:** Fatty acids composition of uterine phospholipids during window of implantation (days 1 to 5 of pregnancy) in intact (control, n=20) and seminal vesicle excised (SVX)-mated (n=20) mice.

	Day 1		Day 2		Day 3		Day 4		Day 5	
	
	Control	SVX-mated	Control	SVX-mated	Control	SVX-mated	Control	SVX-mated	Control	SVX-mated
16:0 (%)	25.24±4.72	25.93±5.46	27.82±4.44	21.56±2.21*	29.48±3.67	24.01±4.65	24.21±3.30	23.22±3.14	21.83±4.43^b, c^	21.38±3.16
16:1 (%)	3.52±0.76	3.17±0.48	3.47±1.03	4.06±0.81	3.72±0.58	4.09±0.52	4.08±1.25	3.89±0.96	3.90±1.39	4.22±0.95
18:0 (%)	24.71±4.58	23.15±4.04	27.00±3.47	23.08±3.20	29.33±3.91	24.45±3.40	24.79±4.95	22.92±3.11	21.03±3.52	23.33±3.53
18:1 (%)	21.40±2.26	22.62±4.93	24.81±3.46	27.00±4.44	24.84±4.18	28.00±4.35	24.20±3.31	27.10±4.06	23.02±4.56	25.15±3.46
18:2 (%)	11.45±2.76	10.70±2.91	7.49±0.76	10.16±1.91	5.29±1.18^a^	8.16±1.94*	10.28±2.80^c^	10.00±1.96	13.81±2.78^b, c, d^	12.06±2.17
18:3 (%)	1.85±0.46	1.77±0.33	1.88±0.17	2.63±0.74	2.46±0.58	2.28±1.04	2.42±0.84	3.04±1.99	2.33±0.40^a^	3.44±1.33
20:4 (%)	12.00±2.63	12.68±2.29	7.54±2.43^a^	11.55±1.51*	4.95±2.99^a, b^	9.00±1.32*	9.82±1.92^a, c^	10.32±2.06	14.20±2.40^b, c, d^	10.40±1.75*
SFA (%)	49.96±2.91	49.08±1.44	54.82±1.82	44.64±1.47*	58.82±4.66^a^	48.46±6.07*	49.00±2.81^c^	46.14±5.53	42.87±1.37^a, b, c^	44.71±6.62
MUFA (%)	24.93±1.93	25.79±5.24	28.28±3.11^a^	31.06±3.77	28.56±4.15^a^	32.09±4.67	28.28±3.73^a^	30.99±4.93	26.91±4.48	29.37±4.21
PUFA (%)	25.31±2.59	25.15±4.06	16.92±1.72^a^	24.34±3.30*	12.70±2.71^a, b^	19.45±3.13*	22.74±2.25^c^	22.86±1.86	30.28±5.20^b, c, d^	25.90±2.66
SFA/UFA (%)	1.00±0.12	0.96±0.05	1.21±0.08	0.80±0.05*	1.45±0.30^a, b^	0.96±0.23*	0.96±0.10^c^	0.87±0.21	0.75±0.04^a, b, c^	0.83±0.22

Data are shown as Mean ± S.D; **SFA =** saturated fatty acids; **MUFA =** mono-unsaturated fatty acids; **PUFA =** poly-unsaturated fatty acids; **SFA/UFA =** ratio of saturated to unsaturated fatty acids; Levene's test confirmed homoscedasticity of variances and two-way ANOVA was used to evaluate interaction between groups and days; Significant difference (p<0.05) in comparison with ^**a**^ Day1, ^**b**^ Day2, ^**c**^ Day3, ^**d**^ Day4 and * between the groups at same day.

We found that levels of linoleic acid (18:2, LNA) and arachidonic acid (20:4, ARA) and consequently PUFA showed a significant decrease from day one to day three of pregnancy. However, their levels increased on days 4 and 5 of pregnancy as these levels were significantly higher than days 2 and 3 of pregnancy (p<0.05). The levels of the omega-6 FA on day five were clearly higher compared to the second, third and fourth day of pregnancy (*p*<0.05). In sharp contrast, the level of saturated FA (SFA) indicated an increase on days two and three compared to day one (p<0.05) and then decreased on the fourth and fifth days of pregnancy. The SFA/PUFA ratio changed similarly but the PUFA levels changed exactly in inverse proportion to the ratio and SFA levels. We observed that the ratio was higher on day three than day one of pregnancy and then started to decrease from the fourth day and reached the lowest level on the fifth day of pregnancy in our experimental window (Table-3). The linolenic (18:3) level did not change significantly during the implantation period and just was statistically lower on day five than day one of pregnancy (*p*<0.05).

To find out possible effects of seminal vesicle secretion on phospholipid FA composition in uterus tissue of mice during the window of implantation, we compared the FA levels between control and SVX-mated groups (data presented in Table-3). Our results demonstrated that effect of seminal vesicle secretion on the composition of the FA was prominent mostly on the second and third day of pregnancy. The levels of LNA on day three were significantly higher in SVX-mated group compared to controls (*p*=0.047). We observed that the levels of ARA and PUFA on the second and third day, were significantly lower in control group compared to the SVX-mated group. In contrast, the levels of SFA and SFA/UFA on those days were higher in control group than the SVX-mated group. Also, the PAM level was significantly higher in control group than the SVX-mated group on the second day of pregnancy (p=0.045). On the fifth day, only the level of ARA showed a significant difference between control and SVX-mated groups (p=0.043, Table-3).

**Figure 2:**
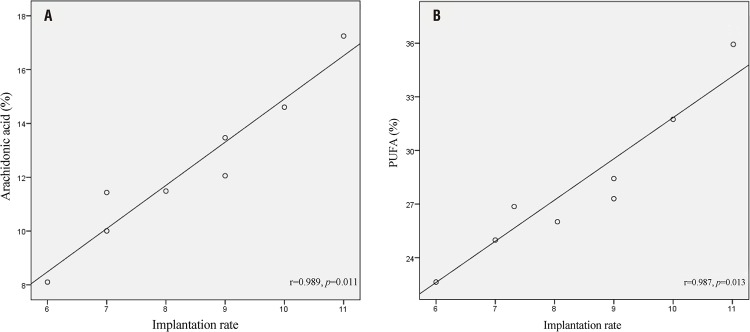
Correlation between implantation rate with a) arachidonic acid (ARA) and b) polyunsaturated fatty acid (PUFA) levels on the fifth day of pregnancy in the control mice.

The implantation rate on day five of pregnancy was significantly higher in control group in comparison with SVX-mated females (9.5±1.29 and 7.25±1.25, respectively; p=0.047). We also evaluated the possible correlation between implantation rate and uterine phospholipid FA levels on the fifth day of pregnancy and found positive correlations between implantation rate and ARA and PUFA levels in control group and not SVX-mated mice (Figure-2).

## DISCUSSION

Our results showed that in control group LNA and ARA, as well as PUFA levels, tended to decrease from first to the third day of pregnancy. It is possible that this happens as a result of the initiation of inflammatory processes following the mating and semen contact with the uterus and consumption of LNA and ARA as the main precursor of PG synthesis (6). Following deposition of semen, inflammatory response has been reported previously in the uterine lumen (16). In accordance with this, we found that the levels of LNA and ARA on the second and third day in the control group were lower than the SVX-mated group; which implies the pivotal role of seminal vesicle secretion in mating-induced inflammation and consequently, uterus phospholipid FA composition. We also found that the levels of LNA and ARA started to increase on days four and five of the pregnancy. Such an increasing slope in the levels of LNA and ARA could increase the availability of PG precursors. In support of this hypothesis, a close association between FA change and PG biosynthesis in endometrium has been reported previously (17). ARA participates in series 2 PG production such as prostaglandin E_2_ (PGE_2_) and prostaglandin F_2_ (PGF_2_); increased levels of PGE_2_ and PGF_2_ has been observed on day five of pregnancy (18). Moreover, previous studies have found rising trends in the expression of enzymes involved in PG synthesis and action in the uterus of mice during the preimplantation period (19). Possible explanation for the observed increase in LNA and ARA levels on days four and five of pregnancy is the presence of an embryo. However, as a limitation of our study it should be mentioned that we evaluated whole uterus and for better clarification future studies should investigate implantation site alone rather than whole uterus. In contrast with LNA and ARA, the PAM and SFA level increased on days two and three and decreased on days four and five of pregnancy. Increase in PUFA and decrease in PAM and SFA levels in total uterine phospholipids on days four and five could possibly augment the membrane lipid fluidity. Since during implantation the membranes of blastocyst and uterus are fused together, such alterations might be in favor of embryo implantation (5). Another reason for reduction of PAM and SFA could be consumption as an energy source, since the importance of FA as an energy source for decidualization has been previously reported (4).

Comparison of the FA composition between the groups showed that LNA and ARA, as well as PUFA levels, were significantly higher and SFA level was lower on the second and third day of pregnancy in mice mated with SVX males compared to the control group. Presence of semen in the female reproductive tract, especially in mice where the semen directly comes in contact with the uterus, could induce inflammatory responses (20). It has been well documented that the immediate response to insemination in the mice is induction of proinflammatory cytokines synthesis in uterine epithelial cells, such as interleukin 6 and 8, macrophage chemotactic protein-1 and granulocyte-macrophage colony-stimulating factor (GM-CSF) (21). Production of PG, especially series 2 is essential for promoting inflammation and their production depends on the supply of precursors such as LNA and ARA. Possible reason for the higher levels of LNA and ARA on days two and three in female mice mated with SVX male is a weaker post-coital inflammatory response in the female, and consequently less LNA and ARA depletion from the pool of membrane phospholipids. In accordance with this explanation, in our recent study we observed lower expression of enzymes responsible for PG biosynthesis in the uterus of mice mated with SVX male, compared to that of the control (12). In the present study, we found a higher level of ARA on implantation day in the uterus of the control group than the SVX-mated group. The increased level of ARA on the day of implantation possibly was observed due to the presence of an embryo. Considering that a higher implantation rate was observed in control than the SVX-mated group, the higher ARA level in the control group could be due to higher implantation rate and embryo numbers.

As we also reported previously (15), there were positive correlations between levels of ARA and PUFA on the day of implantation with the implantation rate in normally mated group. In accordance with this findings, a higher amount of ARA in the uterus of pregnant cows than non-pregnant cows has been demonstrated previously (22). The observed association could be due to the important role of ARA as the precursor of PG which are essential for implantation through inducing angiogenesis and vascular permeability (23). We did not observe the same association in the SVX-mated group, which could be due to disruption of implantation time and/or signaling in this group. Also, increase in ARA and PUFA in the membrane of uterus cells can increase the membrane fluidity and so provoke cell signaling and membrane permeability which can potentially affect embryo-endometrial crosstalk through protein and exosomes/microvesicles mediated signaling during implantation (24). Induction of cell membrane fluidity following increase in PUFA and ARA levels could also affect the endometrial cell- matrix and cell-cell interactions which can potentially affect embryo apposition, adhesion, and invasion. The phospholipid fatty acids (FA) affect the membrane lipid matrix and consequently the mobility, conformation, and function of the membrane proteins (1). On the other hand, successful implantation of the embryo takes place during a specific period known as the window of implantation in which fusion of the blastocyst and uterus cells membranes is an important event. In support of this, essential roles of lipid molecules in mice embryo invasion have been emphasized previously (2).

However, our study was preliminary and it remained to be clarified how the changes in phospholipid FA in the uterus during implantation could play a role in implantation and pregnancy outcome. Besides, the present study evaluated effect of seminal vesicles secretion at implantation window and it needs to be investigated that if it can also affect the pregnancy at post-implantation level or not. Future study could be conducted on phospholipids changes during implantation and implantation period in different cells of the uterus as well as in implantation and non-implantation sites.

## CONCLUSIONS

In conclusion, our results showed that the phospholipid FA composition, especially LNA and ARA levels were changed during the window of implantation in mice uterine tissue. Levels of these FA increased on the day of implantation, while the SFA level decreased. Also, we observed that the seminal vesicle secretion could possibly affect the levels of LNA, ARA, SFA, and PUFA in uterine phospholipids, especially on days 2 and 3 of pregnancy. Considering such changes in uterine FA composition during the implantation period, it could be concluded that FA, especially those participating in PG biosynthesis, may have an important role in early pregnancy. Moreover, seminal vesicle secretion could affect implantation process partly through affecting the uterine fatty acids composition.
